# Evaluation of online dose‐guided adaptive fractionation for intact prostate radiotherapy

**DOI:** 10.1002/acm2.70865

**Published:** 2026-10-14

**Authors:** Gevork S. Gevorkyan, Libing Zhu, Riley C. Tegtmeier, Diego Santos Toesca, Weiguo Lu, Mingli Chen, Yi Rong, Quan Chen

**Affiliations:** ^1^ Department of Radiation Oncology Mayo Clinic Arizona Phoenix Arizona USA; ^2^ Department of Radiation Oncology University of South Florida Morsani College of Medicine Tampa Florida USA; ^3^ Medical Artificial Intelligence and Automation Laboratory UT Southwestern Medical Center Dallas Texas USA

**Keywords:** adaptive, fraction size, Prostate

## Abstract

**Background:**

Dose‐guided adaptive fractionation (DGAF) was proposed as a simpler alternative of adaptive radiation therapy, using organ variation simulation for proof of concept. However, its dosimetric benefit using actual daily anatomy changes in prostate cancer patients remains unclear.

**Purpose:**

The purpose of this study was to retrospectively evaluate the dosimetric benefit and potential risk of DGAF for prostate cancer patients treated with moderately hypofractionated radiotherapy and stereotactic body radiotherapy using daily cone‐beam CT.

**Methods:**

Patients assessed in this study were treated with moderately hypofractionated radiotherapy (MHRT, *n* = 11, 244 total fractions) and stereotactic body radiotherapy (SBRT, *n* = 11, 55 total fractions). The optimizer algorithm computed the desired fractional dose based on favorable or unfavorable anatomical conditions derived from daily imaging, quantified by the sparing factor threshold (μ) and spread (σ). Three methods of estimating subsequent fractionation schemes were explored. The assessment relative to patient‐specific probabilities observed post‐treatment includes the cohort‐based probability (CBP) approach, the individual‐based probability (IBP) approach, and the hybrid approach that begins as CBP and transitions to the IBP after a certain number of fractions. The benefits/risks of DGAF were assessed by calculating the equivalent dose in 2 Gy fractions (EQD2) for both the CTV and the rectum in comparison to regular fractionation.

**Results:**

DGAF demonstrated potential dosimetric benefits for intact prostate patients. The average and standard deviation of the sparing factor was 0.51 ± 0.11 for MHRT patients and 0.42 ± 0.04 for SBRT patients treated with endorectal‐balloon. The hybrid approach for estimating the patients’ threshold μ and spread σ outperformed other approaches assessed for both cohorts. The highest dosimetric benefit was retrospectively seen in MHRT patients where a hybrid approach were used, showing equal or decreased D15% EQD2.

**Conclusion:**

DGAF demonstrated considerable rectum sparing potential, and the benefits increased with longer fractionation and advanced approaches toward subsequent fraction estimation.

## 1 INTRODUCTION

The next generation approach for radiation therapy (RT) will include daily personalized treatments accounting for patients’ daily anatomy changes.^1^, ^2^ Commercial platforms incorporate integrated workflows to account for daily anatomical changes via online adaptation,^3^, ^4^, ^5^ which are associated with high workflow cost and extended treatment times.^6^, ^7^ Such adaptive radiotherapy (ART) platforms include the computed tomography (CT)‐based Ethos platform (Varian Medical Systems, Palo Alto, CA, USA),^8^, ^9^ and the magnetic resonance imaging (MRI)‐based Unity (Monaco‐Elekta AB, Stockholm, Sweden)^10^, ^11^, ^12^ and MRIdian (ViewRay Inc., Oakwood Village, Ohio, USA).^13^ Recent clinical studies have reported dosimetric improvements and limited rates of acute GI and GU toxicity following adaptive MR‐guided radiotherapy, albeit definitive reductions in toxicity have yet to be established.^5^, ^14^ However, these commercial platforms are not widely available because of the significant capital investment required. Additionally, plan re‐optimization may prolong the duration that the patient remains on the treatment table, ranging between 45 min–1 h,^15^ increasing the burden to patients, the staff, and the clinical schedule.

Dose‐guided adaptive fractionation (DGAF) was proposed as a simplified approach to account for daily anatomical changes, leveraging the variation in a patient's sparing factor on each fraction to adapt the fractional dose while maintaining the same physical dose or biological equivalent dose (BED) by the end of treatment.^16^, ^17^, ^18^ The daily fraction dose was optimized within predefined minimum and maximum dose limits that depend on the remaining number of fractions, ensuring that the total prescription dose can still be achieved regardless of previous adaptations. Fractions with favorable anatomy would be given a higher dose, whereas fractions with unfavorable anatomy would be given a lower dose, with the aim of reducing cumulative organ‐at‐risk dose while maintaining the prescribed total target dose. Previous studies demonstrated the overall organs at risk (OARs) sparing achievable through DGAF—one study on patients treated with five‐fraction regimens to abdominal tumors assessed the dose to 1 cubic centimeter of an OAR divided by dose exceeding 95% of the planning target volume (PTV) and showed that overall sparing was possible.^19^ This study assessed a dose‐volume‐histogram (DVH) akin to the maximum dose of the OAR normalized by the uniform dose applied to the PTV. In a separate study of conventionally fractionated RT for definitive treatment of prostate cancer patients, overall sparing was possible when looking at the accumulated delivered dose mapping.^20^ The method used in that study was more complex, and the details of their approach were not completely described. Overall, prior studies have not properly defined how to incorporate alternative OAR DVH constraints.^21^, ^22^ More commonly, previously developed algorithms used a strictly individual‐based probability approach to derive patient statistics, which relied on only two data points at the beginning of fractionation—one from the CT sim and one from the CBCT imaging prior to first fraction delivery. Additionally, simple algorithmic approaches were applied in predicting the variation of sparing factors in subsequent fractions without exploration of the optimal approach for doing this on independent patient cohorts. Therefore, questions remain regarding the practical application and clinical implementation of DGAF during treatment. The concise dosimetric benefit of the DGAF approach to actual patient treatments has been left wanting.

In this work, the advanced probabilistic DGAF algorithm established by other studies was used for deciding the dose delivered each fraction.^16^, ^17^ In theory it is possible to choose any size fraction ranging from no dose to the full remaining prescription dose; however, delivering no dose and potentially doing quality assurance on a variable number of plans is not clinically practical. In the outline of DGAF presented here, we require preparing one plan with two to three normalizations prepared for delivery. We retrospectively assessed the use of DGAF on patients treated for prostate malignancies using their daily OAR changes based on on‐board kilovoltage iterative cone beam CT (iCBCT) imaging.^23^, ^24^, ^25^, ^26^, ^27^, ^28^, ^29^ We assessed the variation of sparing factors for a cohort treated with moderately hypofractionated radiotherapy (MHRT) and for a second cohort treated with stereotactic body radiotherapy (SBRT). We established a novel approach utilizing both cohort‐based data and individual patient data for optimal dose‐guidance. The overall dosimetric benefit and potential risks of DGAF applied on clinical data was quantified and reported.

## 2 METHODS

### 2.1 Patient descriptions and data

Upon approval from the Institutional Review Board (IRB # 22–001608), 22 prostate cancer patients with a total of 299 fractions, treated definitively with volumetric modulated arc therapy (VMAT) radiation therapy and daily kV iCBCT‐based image guidance, were used in this study. Among them, 11 were treated with MHRT (*n* = 8 given 60 Gy in 20 fractions, *n* = 3 given 70 Gy in 28 fractions), and the others were treated with SBRT (*n* = 11 given 36.25 Gy in 5 fractions). Patient simulation was the same for both patient cohorts. No rectal spacer was used in MHRT. An endorectal‐balloon (ERB) was used among patients treated with SBRT. Selection criteria excluded patients with prosthetic hip arthroplasty due to unfavorable iCBCT image quality for dose assessment.

Treatment plans were designed with a normalization of 100% prescription dose to 95% of the PTV, covering CTV D98% > 100%, maintaining both bladder and rectum D0.03cc < 105%, V90% < 10%, and V65% < 50%, based on our institutional protocol. The per‐fraction CTV and rectum contours were obtained using a deep‐learning‐based pelvis iCBCT contouring model,^30^, ^31^, ^32^, ^33^ and were validated and corrected by certified physicians. The RT plan was propagated to the daily iCBCT image of every fraction for each patient within the Eclipse treatment planning system.

### 2.2 Defining and assessing sparing factors

The DVH of relevant OARs can quantify the sparing achieved in a treatment plan.^34^, ^35^, ^36^, ^37^, ^38^ They are optimized during planning based on dose‐volume relationships to radiation‐induced gastrointestinal (GI) and genitourinary (GU) toxicity previously reported in the literature. During treatment, anatomical changes, including variations in organ filling, patient positioning, and tumor geometry, may cause the delivered doses to both the OARs and the target to vary from fraction to fraction. Therefore, the OAR dose and target dose are defined separately for each treatment fraction *i*. The OAR dose can be expressed as a fraction of the target dose:

(1) $$\delta_{i}=d_{i}^{OAR}/d_{i}^{target}$$

where $d_{i}^{OAR}$ is the fraction‐specific OAR dose metric, $d_{i}^{target}$ is the target dose, and $\delta_{i}$ is the OAR sparing factor. The target dose $d_{i}^{target}$ is assumed to equal the prescribed dose in each fraction as image‐guided radiation therapy (IGRT) maintains accurate target localization and adequate target coverage. The OAR dose, $d_{i}^{OAR}$, may be represented by any dose metric sensitive to anatomical changes. This representation may be a single dose metric, a weighted combination of multiple dose metrics, or even a combination of metrics from different OARs. For simplicity we chose to assess the *D*_15%_ dose to rectum, a dose metric indicative of rectum tolerance,^22^, ^39^ as our $d_{i}^{OAR}$ to calculate the sparing factor for optimization.

### 2.3 DGAF based on clinical patient sparing factors

An advanced probabilistic DGAF algorithm was used for deciding the dose to be delivered each fraction.^16^, ^17^ For feasibility and simplicity in this retrospective application on clinical patient sparing factors, we assumed two treatment normalization options for delivery to patients using an even number of fractions–150% and 50% of the original fraction dose as the upper and lower bounds, respectively. As an example, for 60 Gy in 20 fractions, 60 Gy = 10 fx [1.5 Gy/fx] + 10 fx [4.5 Gy/fx]. For patients with an odd number of fraction treatments, such as five‐fraction SBRT, the original fraction dose is included as a third option to fulfill the physical prescription dose. As an example, for 36.25 Gy in 5 fractions, 36.25 Gy = 2 fx [3.63 Gy/fx] + 1 fx [7.25 Gy/fx] + 2 fx [10.87 Gy/fx].

The DGAF algorithm requires the estimation of the distribution of anatomical configurations in subsequent fractions to select higher dose on anatomically favorable days and lower dose on anatomically unfavorable days. The algorithm therefore requires an input of distribution parameters: mean (µ) and standard deviation (σ)—this is notably insensitive to the specific form of the distribution.^16^ The challenge lies in having a reliable and accurate approach for estimating possible subsequent fractions. One simple approach which has not been previously examined would be a CBP approach, where the patient's planning CT sparing factor is used as the threshold value µ and the average variation of patient sparing factors within the same cohort is used as spread value σ.

The performance of DGAF decreases when the actual distribution differs from the prediction model, as shown in simulation.^16^ This CBP approach used on the first fraction is a reasonable starting point; however, there can be more than one justifiable approach to estimate the shape of this distribution of sparing factors in the first fraction and in subsequent fractions. An IBP approach was adopted in a prior study.^19^ For IBP, the threshold and spread values are continuously updated based on patient daily iCBCT imaging:

(2) $$\mu_{i}=\frac{1}{f+1}\;\sum_{i=0}^{f}\delta_{i},$$

(3) $$\sigma_{i}=\sqrt{\frac{1}{f+1}\sum_{i=0}^{f}{\left(\delta_{i}-\mu_{i}\right)}^{2}}$$

where the fraction threshold value µ_i_ and fraction spread value σ_i_ are the average and standard deviation of the specific patient's sparing factor up to the current fraction (including the planning CT value).

Equation 2 is the threshold value for delivering higher or lower fraction doses, whereas equation 3 acts as a risk assessment value particularly important toward the end of fractionation. However, in the first few fractions there are only two data points, one from the planning CT and one from the iCBCT imaging of the first fraction. Using such few data points to assess the threshold or spread in the probability might not be a sound approach for every cohort. Hence, we propose a hybrid approach using CBP on the first several fractions before switching to IBP for the remaining fractions. The hybrid approach was tested by switching on the 2^nd^, 3^rd^, 4^th^, 5^th^, 6^th^, and 7^th^ fractions, respectively. We compared the CBP, IBP, and hybrid approaches by assessing the accuracy of the estimated probability distributions with the patient sparing factor probability known at the end of fractionation retrospectively.

### 2.4 Assessment of dose‐guided adaptive radiation therapy benefit

We defined the dose received by the rectum as the product of the sparing factor and the physical dose delivered in the fraction as seen in equation 1. The fraction dose was used to assess the dose to the CTV. The BED model^40^ was applied to calculate the EQD2. The radiosensitivity ratio used in this study was *α/β* = 3 for normal tissue and *α/β* = 3 for the CTV.^41^

The cumulative EQD2 ratio between the rectum and CTV was used as a surrogate measure of the biological dose delivered to the rectum relative to the target following DGAF:

(4) $$\Delta_{r/c}=d_{r}\;\left(\mathrm{reg}\right)-d_{r}\left(\mathrm{opt}\right),$$

where d_r_(reg) and d_r_(opt) are the cumulative rectum‐to‐CTV EQD2 ratios obtained with conventional fractionation and DGAF, respectively. Although the rectum dose for an individual fraction is given by the sparing factor multiplied by the prescribed fraction dose, the reported ratio is calculated from the cumulative EQD2 values over the entire treatment course. Because EQD2 depends nonlinearly on dose per fraction, redistributing the prescribed dose across fractions changes both the cumulative CTV and rectum EQD2, even when the total prescribed physical dose to the CTV is maintained. A reduction in the cumulative rectum‐to‐CTV EQD2 ratio therefore indicates a lower biological dose to the rectum relative to the target with DGAF. Since unfavorable sequences of sparing factors near the end of treatment may limit the algorithm's ability to adapt the remaining fractions while satisfying the prescribed cumulative dose, we also assess if there were patients where the rectum‐to‐CTV EQD2 ratio actually increased along with the magnitude of the increase. We will assess the real patient results (Equation 4) for varying approaches on each cohort—the CBP approach, hybrid approach starting from some fraction “i”, and lastly the IBP approach.

## 3 RESULTS

### 3.1 Patient sparing factors

The sparing factor of every fraction for all patients, as defined in equation 1 using rectum D15% dose, are shown in Figure 1. The planning CT values are shown in red circles.

**FIGURE 1:**
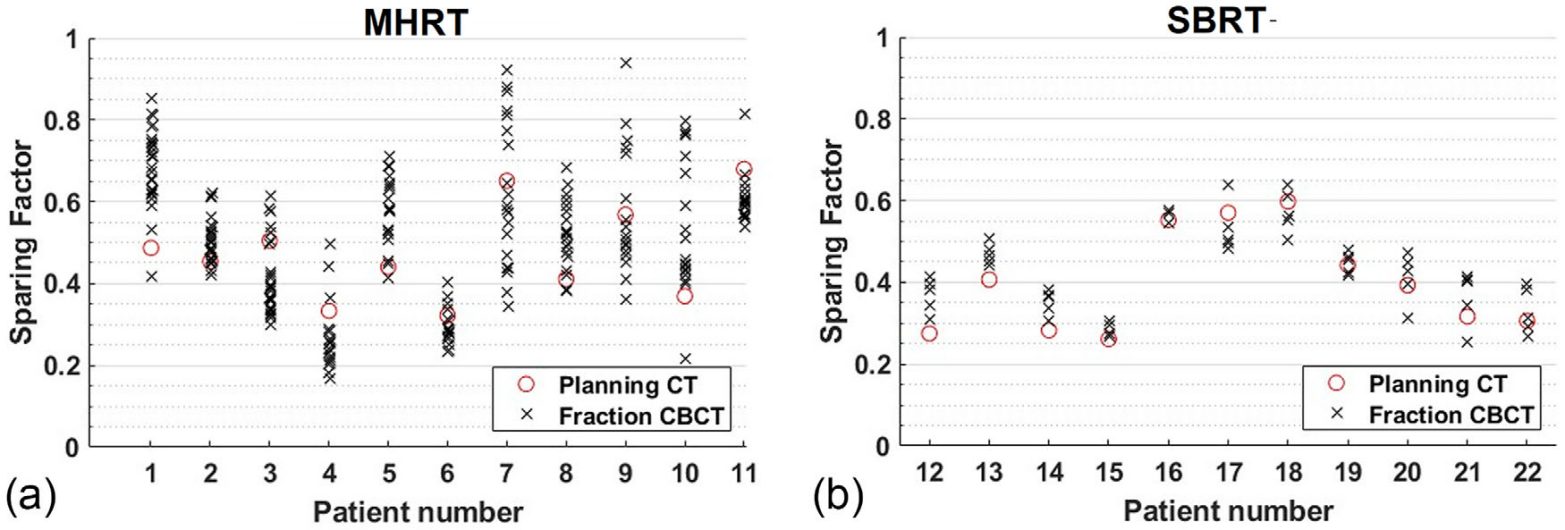
Rectum D15% sparing factor shown in 1‐a for MHRT patients (1–11) and in 1‐b for SBRT patients (12–22). The sparing factor from the planning CT (red, circle) and the remaining fractions (black, cross) of each patient are marked for visual comparison.

The planning CT sparing factor for each patient was observed to be randomly distributed among the values found throughout the course of treatment. The average and standard deviation of the sparing factor for MHRT patients was 0.51 ± 0.11. The SBRT patients (with ERB) had an average and standard deviation sparing factor of 0.42 ± 0.04. Four out of the eleven MHRT patients had a final fraction sparing factor larger than the individual patient's average, and six out of the eleven SBRT patients had a final fraction sparing factor larger than the individual patient's average.

A plot of the agreement of the patient‐specific treatment variation is shown for each fraction in Figure 2. An error of zero represents the perfect case, where the estimated mean or standard deviation exactly matches the value calculated after all treatment fractions have been completed. The dashed blue lines indicate the CBP approach value. The error in estimating the patient average sparing factor using IBP reduces below CBP after the first fraction for MHRT patients, whereas it is immediately better in the first fraction for the SBRT patients. This is because the sparing factors varied between patients in both cohorts, however the SBRT cohort sparing factor varied less within an individual patient throughout treatment. In comparison to using CBP for estimating the standard deviation, using IBP has less error after several fractions for MHRT patients and after the first fraction for SBRT patients.

**FIGURE 2:**
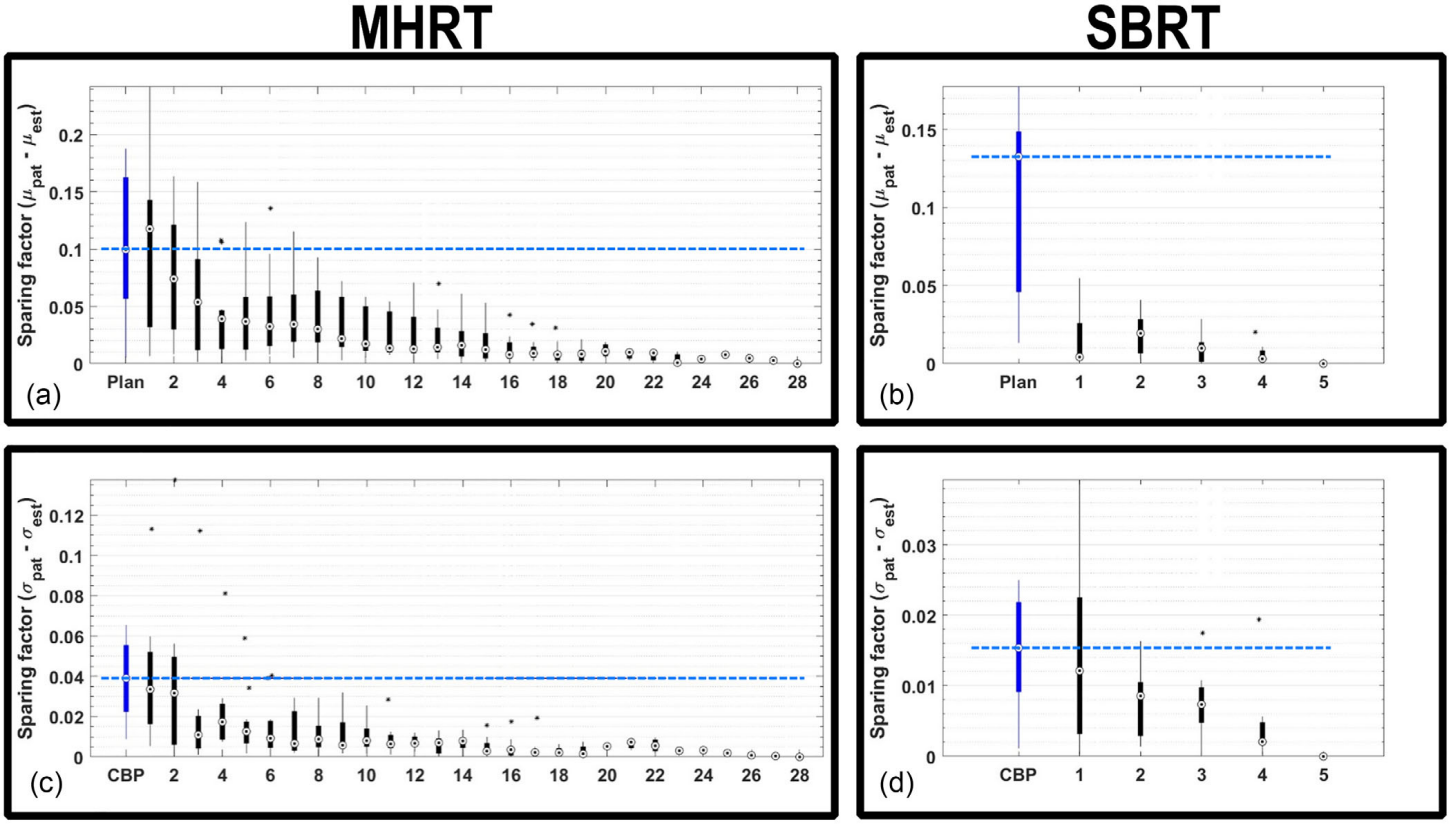
Comparison of per‐fraction relative error in sparing factor estimation using IBP‐ and planning CT‐based approaches, where a zero value indicates the exact patient distribution by the end of fractionation. The dashed lines represent the respective CBP approach value as reference.

### 3.2 Dose adaptation and sparing factor probability estimation method results

Retrospectively, all patients were assessed a higher EQD2 dose to the CTV with DGAF relative to regular fractionation. A box‐and‐whisker plot of the real patient retrospective results (Equation 4) on rectum D15% with DGAF are shown in Figure 3. Both the MHRT and SBRT patient results are shown using different approaches on the horizonal axis—the CBP approach is centered around the planning CT sparing factor, a hybrid approach which begins as CBP and transitions to IBP at a given fraction number “i” (denoted as H_i_), and lastly the IBP approach. The patient‐specific nature of the IBP and hybrid approaches of DGAF proved to be an improvement over the CBP approach. The hybrid (H_3_ and H_4_) approaches of DGAF showed equal or reduced rectum D15% EQD2 ratio for the entire MHRT cohort. The SBRT patients generally yielded less results compared to MHRT regardless of approach used on DGAF, with much of the cohort yielding lower rectum D15% EQD2 ratio and a few patients cohort yielding a higher ratio.

**FIGURE 3:**
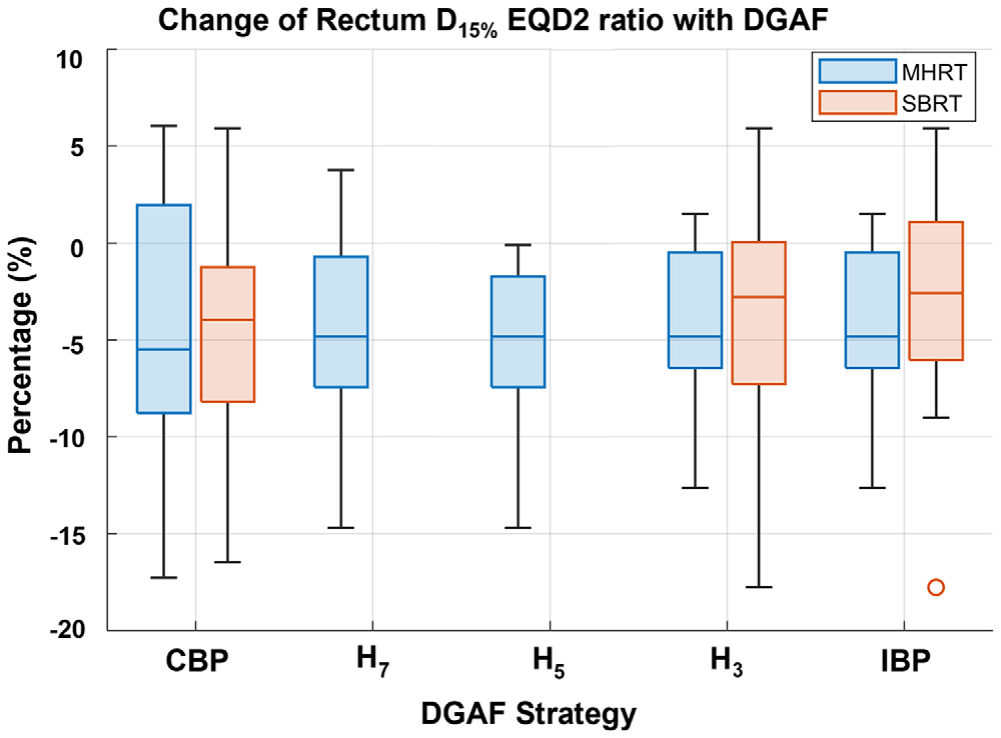
Percentage change in rectum EQD2 ratio (Equation 4) with DGAF. The approaches used were CBP, hybrid at fraction “i” (H_i), and IBP from the start of fractionation. A negative percentage indicates a reduction in rectum D15% with DGAF.

## 4 DISCUSSION

Adaptive fractionation provides patient‐specific care addressing the day‐to‐day changes in patient anatomy, which is an improvement over the conventional “one‐plan‐fits‐all” approach. Although in principle it is possible to vary the fractionation into a continuous spectrum of dose, the clinical implementation would require nearly one‐hour long workflows similar to that of some commercial online ART platforms. In comparison, DGAF requires preparing two or three plans for delivery and 4–7 min extra online time for AI‐based iCBCT contouring and DVH calculation for daily sparing factor calculation.^33^

The two factors necessary for prediction are the threshold value of the sparing factors and the spread of the sparing factors for the specific patient. The threshold value determines whether to deliver lower or higher dose for a fraction, and the spread helps predict the likelihood of future fraction sparing factors. It is possible to go with an IBP, CBP, or hybrid approach for either of these values, and we have demonstrated different approach combinations in our results. Other studies have noted that the probability used in DGAF is crucial for maximizing the sparing potential and decreasing the risk of potentially delivering higher dose to OARs.^16^ To the knowledge of the authors, no studies have assessed the approach of adaptively changing the distribution estimation during fractionation for optimizing the sparing factor prediction accuracy. The threshold value is important for daily decision making, and adopting a hybrid approach early in treatment was adopted and showed successful dose optimization. The spread for a specific patient is not well determined early on because it can vary in the first few fractions, but it can potentially vary more from patient to patient even within the same cohort, so a hybrid approach is optimal for determining the spread in both cohorts. We observed that all approaches used for SBRT patient cohort yielded similar results due to the smaller spread in sparing factors with the use of ERB, although this treatment modality had too few fractions to experience the largest benefit from DGAF as presented in other works^21^, ^22^, ^23^, ^24^ and confirmed in our observations. These two observations, regarding sparing factor variation and benefit through increased fractionation, agree with the DGAF simulation and theory established by others.^16^, ^17^

Studies have investigated constraining the algorithm to deliver the total BED,^17^ however the physician's prescription is still based in physical dose because the adoption of BED is still not widespread.^42^, ^43^, ^44^, ^45^ Therefore, the DGAF algorithm was constrained to deliver the total physical prescription dose using two dose options in the present study, and for simplicity we divided the fractionation into equal parts of lower and higher doses as described in section 2.3. However, there might be other fractionation solutions beyond the one that we applied in this study that could enhance the results. For example, it's possible to give a 40‐fraction treatment where 4/7× fraction dose delivered in 35 of the fractions and 4× fraction dose is delivered in 5 of the fractions. As an example, for 72 Gy in 40 fractions, 72 Gy = 35 fx [4/7 * 1.8 Gy/fx] + 5 fx [4 * 1.8 Gy/fx]. In that case, the physical dose would again be the same, but the BED would be higher than the approach used in this study due to the larger upper limit of fraction doses. This may lead to further improved OAR sparing depending on the dose distribution variations.

When testing the proposed algorithms retrospectively using clinical patient data, we observed appreciable sparing potential for MHRT patients and some sparing potential for SBRT patients. The cumulative EQD2 ratio (Equation 4) was intended as an initial indication of dosimetric benefit with DGAF rather than a direct estimate of tumor control probability (TCP) or normal tissue complication probability (NTCP). This is a limitation of our study, and future work will be done to evaluate the impact of the proposed approach using established TCP/NTCP models. Some hybrid approaches (H_3_ and H_4_ on Figure 3) on the MHRT cohort showed only equal or reduced rectum D15% EQD2 ratio, however a limited number of patients were assessed, therefore no definitive conclusions can be made on the exact optimal approach. This work assesses a limited number of patients, and a larger pool of patient data is necessary for a broader study, but that is subject to the availability of iCBCT imaging with appropriate structures and delineated targets.

Although SBRT patients showed some potential dosimetric benefit, there was always some dosimetric risk regardless of approach. Too few fractions meant there was little room for algorithmic prediction of anatomic variation, which is critical for DGAF to produce improved OAR sparing. In addition, with approaches such as ERB and/or rectal spacer, the variability of the rectum dose is greatly limited. This also reduced the opportunities that the DGAF can leverage. As reflected in our results, SBRT treatment is less suitable for DGAF.

Online ART is expected to provide greater dosimetric benefit than DGAF because it fully replans on the new patient anatomy, whereas DGAF only renormalizes the original treatment plan. On the other hand, DGAF is far less resource intensive as it only selects a pre‐scaled plan to deliver. In this study, we observed that DGAF can still provide a dosimetric benefit over regular fractionation, improving the rectum D15% sparing in the majority of cases. To achieve the rectum sparing reported in this study, the clinical practice would still need to implement advanced motion monitoring tools^46^, ^47^, ^48^, ^49^ and appropriate target volume margins^50^ used for planning. This study was limited to assessing one DVH metric for one selected OAR as an initial attempt to outline a clinical approach to DGAF, but other OARs were not accounted for and will be investigated in future work. A direct comparison between the sparing achieved with daily replanning and with DGAF will be explored in a future study.

## 5 CONCLUSION

DGAF demonstrated considerable potential rectum sparing for a large majority of intact prostate patients treated with MHRT, showing dosimetric benefits on patients with longer fractionation treatments. The results were not compelling for use with SBRT due to too few benefits with short fractionation. Using a hybrid approach to estimate the threshold and spread of sparing factors yielded best results for OAR sparing. The DGAF method described in this work may provide a practical framework for accounting for daily anatomical variations and individualizing fraction‐level dose adaptation.

## AUTHOR CONTRIBUTIONS

Quan Chen conceived the presented idea and designed the experiments with Yi Rong and Libing Zhu. Gevork S. Gevorkyan, Libing Zhu and Quan Chen carried out experiments and analysis of the results. Libing Zhu, Riley C. Tegtmeier and Gevork S. Gevorkyan contributed to the data preparation. Diego Santos Toesca, Yi Rong and Quan Chen provided interpretation of the result. Weiguo Lu and Mingli Chen provided the DGAF calculation code and instructions for the study. Gevork S. Gevorkyan and Quan Chen took the lead in writing the manuscript and addressing reviewer comments. All authors provided critical feedback and helped shape the research, analysis and manuscript.

## CONFLICT OF INTEREST STATEMENT

Quan Chen is co‐founder of Carina Medical LLC. All other authors have no conflict of interest to declare.

## ETHICS STATEMENT

The study is approval by the Institutional Review Board under IRB # 22–001608.

## Data Availability

Research data are stored in an institutional repository and will be shared upon request to the corresponding author.
